# A Prototype Microwave System for 3D Brain Stroke Imaging

**DOI:** 10.3390/s20092607

**Published:** 2020-05-03

**Authors:** Jorge A. Tobon Vasquez, Rosa Scapaticci, Giovanna Turvani, Gennaro Bellizzi, David O. Rodriguez-Duarte, Nadine Joachimowicz, Bernard Duchêne, Enrico Tedeschi, Mario R. Casu, Lorenzo Crocco, Francesca Vipiana

**Affiliations:** 1 Department of Electronics and Telecommunications, Politecnico di Torino, 10129 Torino, Italy; jorge.tobon@polito.it (J.A.T.V.); giovanna.turvani@polito.it (G.T.); david.rodriguez@polito.it (D.O.R.-D.); mario.casu@polito.it (M.R.C.); 2 Institute for the Electromagnetic Sensing of the Environment, National Research Council of Italy, 80124 Naples, Italy; scapaticci.r@irea.cnr.it (R.S.); crocco.l@irea.cnr.it (L.C.); 3 Department of Electric Engineering and Information Technologies, University of Naples Federico II, 80125 Naples, Italy; gbellizz@unina.it; 4 Group of Electrical Engineering-Paris (GeePs), CNRS, CentraleSupélec, Université Paris-Sud, Univ. Paris-Saclay, Sorbonne Univ., 91190 Gif-sur-Yvette, France; nadine.joachimowicz@paris7.jussieu.fr; 5 Laboratoire des Signaux et Systèmes (L2S), Université Paris-Saclay, CNRS, CentraleSupélec, 91190 Gif-sur-Yvette, France; bernard.duchene@l2s.centralesupelec.fr; 6 Department of Advanced Biomedical Sciences, University of Naples Federico II, 80131 Napoli, Italy; enrico.tedeschi@unina.it

**Keywords:** microwave imaging, brain stroke, monitoring, antenna array

## Abstract

This work focuses on brain stroke imaging via microwave technology. In particular, the open issue of monitoring patients after stroke onset is addressed here in order to provide clinicians with a tool to control the effectiveness of administered therapies during the follow-up period. In this paper, a novel prototype is presented and characterized. The device is based on a low-complexity architecture which makes use of a minimum number of properly positioned and designed antennas placed on a helmet. It exploits a differential imaging approach and provides 3D images of the stroke. Preliminary experiments involving a 3D phantom filled with brain tissue-mimicking liquid confirm the potential of the technology in imaging a spherical target mimicking a stroke of a radius equal to 1.25 cm.

## 1. Introduction

Brain stroke is one of the main causes of permanent injury and death worldwide, with an incidence of over 5 million annual deaths [1]. Since prompt intervention (such as the administration of specific drugs that can affect the acute stage of the stroke) can significantly improve the prognosis, a rapid diagnosis of the disease and continuous monitoring after its onset represent important clinical goals.

Currently, the most effective tool for brain stroke diagnosis is the imaging-based diagnostics performed in an emergency department after the recognition of stroke-like symptoms. In this respect, magnetic resonance imaging (MRI) or X-ray based computerized tomography (CT) are the clinically adopted techniques. However, although they are continuously evolving, these technologies still have several limitations. In particular, despite its high resolution and accuracy, MRI is not widely available in emergency settings and is therefore actually used only as a secondary diagnostic tool [2,3]. On the other hand, non-contrast CT may be limited by the fact that the early signs of ischemia may not be easily recognizable by non-experienced personnel. Moreover, due to the use of ionizing radiation, CT is not suitable for repeated examinations, which are especially useful for post-acute monitoring purposes. Furthermore, both MRI and CT equipment is bulky and so not currently suited for ambulance use or as bedside devices.

The above circumstances have led to increased interest in the development of different diagnostic imaging techniques [3]. Among others, microwave imaging (MWI) [4] has emerged as a complementary technique which is able to address the different needs arising in stroke diagnosis and management, namely the early—possibly prehospital—diagnosis of the kind of stroke (ischemia or hemorrhage), bedside brain imaging and continuous brain monitoring for stroke in the post-acute stage. MWI takes advantage of the different electric properties (electric permittivity and conductivity) that human tissues exhibit at microwave frequencies depending on their kind (e.g., blood versus gray or white matter) and pathological status. These differences permit a functional map of the inspected anatomical region to be obtained. The benefits of MWI mainly stem from the non-ionizing nature of microwave radiation and the reduced intensity required to obtain reliable imaging (at an intensity comparable to that currently used for mobile phones), which make it completely safe and suitable for repeated applications. Moreover, MWI technology is cost-effective and benefits from a reduced size, as it makes use of miniaturized, low-cost, off-the-shelf components that are available in the microwave frequency range for signal generation and acquisition [5] and low-cost accelerators to speed up processing [6].

Recently, several MWI devices and prototypes have been proposed [7,8,9,10,11,12,13,14,15,16,17]. Among them, the two most prominent examples (which are already being tested on humans) are the Strokefinder, developed by Medfield Diagnostics [7,8], and the EMTensor BrainScanner [10]. The Strokefinder is a device which aims to discriminate between ischemic and hemorrhagic strokes in the early stage of patient rescue, based on an automated classification which is carried out by comparing the measured data to a database (obtained by data collected from already examined patients). This device is characterized by its very simple and compact hardware, consisting of a small number of printed antennas mounted on a support that can be adapted to the patient’s head. Some initial clinical trials have been reported for the Strokefinder [7], but it should be remarked that it does not provide images; thus, its intended role is to complement standard imaging tools. The EMTensor BrainScanner aims to perform brain stroke tomography. The system is characterized by a high complexity, as it consists of a large number of radiating elements (177 truncated waveguides, loaded with ceramic material of appropriate permittivity [10]), which considerably increase its cost and size, thus reducing to some extent the advantages of its use. In addition, the image reconstruction task involves the processing of a considerable amount of measured data and has to face the pitfalls of dealing with a non-linear and ill-posed inverse problem. This entails long elaboration times and possibly results in false solutions; i.e., producing images which fulfill the underlying optimization but are different from the ground truth.

In this paper, we describe the realization, characterization and initial experimental validation of a prototype device representing a different approach to dealing with a still open issue in stroke management; that is, continuous monitoring during the hospitalization of the patient in order to evaluate the effectiveness of the administered therapies [18]. This specific application aims to image only a “small” variation occurring in the brain and not its overall structures and features. As a consequence, it is possible to keep the device complexity low, and therefore also its size and cost, as well as to rely on the Born approximation to model the scattering phenomenon, thus enabling reliable real-time imaging. Accordingly, the proposed device is based on the low-complexity architecture designed with the rigorous procedure as described in [18]. Moreover, it adopts a differential imaging approach, where data gathered at two different acquisition times are processed [19] with simple and fast imaging algorithms based on the distorted Born approximation [18,19].

The proposed system provides 3D images of the head by relying on data measured through an array of 24 printed monopole antennas organized as an anatomically conformal shape mimicking a wearable and adaptable helmet. Each antenna is enclosed in a box of graphite-silicon material, acting as the coupling medium, and connected to a two-port vector network analyzer (VNA) through a 24×24 switching matrix, which allows the whole differential scattering matrix required for imaging to be acquired. The use of a semi-solid matching medium is a distinct feature of the system which allows for an increased simplicity of operation and repeatability, as compared to other arrangements that make use of a coupling liquid [10]. Finally, as detailed below, the device presented here is equipped with a “digital twin” based on a proprietary electromagnetic (EM) solver that allows us to properly characterize and foresee its behavior, as well as to provide the building blocks needed for the imaging.

In the following sections, the different components of the device are described and discussed, and a first experimental assessment on an anthropomorphic head phantom is presented. This phantom consists of a plastic shell with the shape and size of a human head, which is filled up with a homogeneous material whose dielectric properties are equal to an average value of the properties of the different tissues present in the brain. The reported experimental results provide an initial demonstration of the capabilities of the developed device.

It is worth noting that the presented system is not the only example of a low-complexity device for brain imaging, as other devices, using a low number of antennas (8–16) arranged in a circular array, have been proposed [12,13,14,15,16]. However, these devices only provide 2D maps of the transverse cross-section of the head in the array plane, whereas the device herein presented provides a full 3D image of the head.

## 2. Material and Methods

### 2.1. Three-Dimensional Microwave Imaging System Design

In this section, we present the choices made in the design of the proposed imaging system (i.e., the operating frequency, coupling medium, antenna number and arrangement).

The operating frequency as well as the properties of the selected coupling medium were set according to previous findings obtained from theoretical formulations and experimentally validated [20] and [4] (Chapter 2). In particular, a working frequency of around 1 GHz and a coupling medium with a relative permittivity of around 20 were determined to be optimal and therefore chosen for the realization of the hardware. As all the simulations and measurements were made at 1 GHz, this operating frequency will be considered as implicit in the following.

To design the layout of the array of antennas (the number and position of the radiating/measuring elements), a recently proposed rigorous procedure was adopted [18,21]. This procedure is based on the analysis of the spectral properties of the discretized scattering operator [22], assuming the antennas to be located on a surface conformal to the head (a helmet) and taking into account the dynamic range and signal-to-noise ratio (SNR) of the adopted measurement device as well as the actual size of the antennas. The result of this study allowed us to identify a 24-element array as the suitable candidate to perform the desired imaging task while keeping the system complexity as low as possible. The expected performances were confirmed by a preliminary numerical analysis [18].

As far as the choice of the imaging algorithm was concerned, the key aspect was that the targeted application was to monitor the time evolution of the stroke. Hence, a differential approach was a suitable choice [18,23]. In particular, the input data of the imaging problem, denoted as ΔS in the following, were represented by the difference between the scattering matrices measured at two different times, while the output was a 3D image showing the possible variation of the electric contrast of the brain tissues—i.e., Δχ—occurring between these two different times. The differential electric contrast function Δχ is defined as $\Delta \epsilon /\epsilon_{b}$, where $\epsilon_{b}$ is the complex permittivity of the non-homogeneous background at the reference instant (e.g., a map of the brain at the first diagnosis) and Δϵ is the complex permittivity variation between the two time instants.

Since the contrast variation Δχ was localized in a small portion of the imaging domain, it was possible to take advantage of the distorted Born approximation [22], so that a linear relationship held between ΔS and Δχ:

(1) $$\Delta S\left(r_{p},r_{q}\right)=S\left(\Delta \chi \right),$$

where S is a linear and compact integral operator, whose kernel is $-j\omega \epsilon_{b}/4\;E_{b}\left(r_{m},r_{p}\right)\cdot E_{b}\left(r_{m},r_{q}\right)$, with $r_{m}\in D$, $r_{p}$ and $r_{q}$ denote the positions of the transmitting and receiving antennas, and $r_{m}$ shows the positions of the points in which the imaging domain *D* is discretized. $E_{b}$ is the background field in the unperturbed scenario; that is, the field radiated inside the imaging domain by each element of the array. The symbol “·” denotes the dot product between vectors, ω=2πf is the angular frequency, and *j* the imaginary unit.

As a reliable and well-established method to invert (1), we exploit the truncated singular value decomposition (TSVD) scheme [22], which allows us to obtain the unknown differential contrast function through the explicit inversion formula:

(2) $$\Delta \chi =\sum_{n=1}^{L_{t}}\frac{1}{\sigma_{n}}\langle \Delta S,\left[u_{n}\right]\rangle \left[v_{n}\right],$$

where $\sigma_{n}$, $\left[u_{n}\right]$ and $\left[v_{n}\right]$ are the singular values and the right and left singular vectors of the discretized scattering operator S, respectively. $L_{t}$ is the truncation index of the SVD, which acts as a regularization parameter and was chosen to obtain a good compromise between the stability and accuracy of the reconstruction [22].

### 2.2. Three-Dimensional Microwave Imaging System Realization

**Figure 1:**
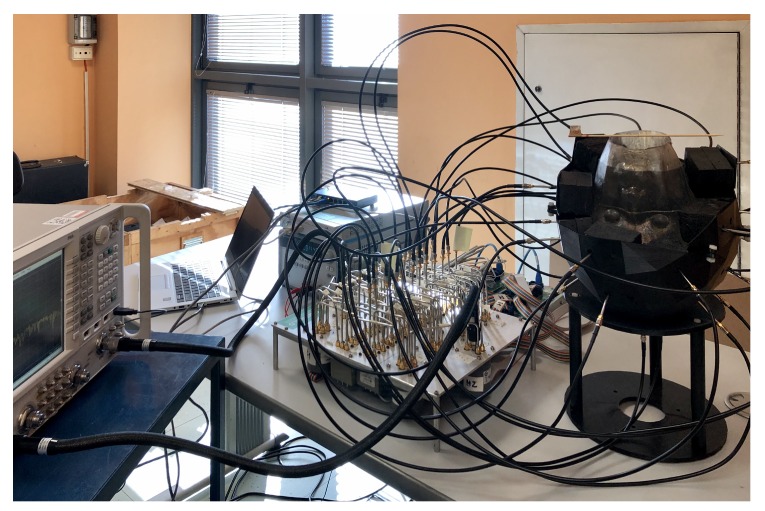
Overview of the realized 3D microwave imaging system prototype. From left to right: vector network analyzer (VNA), switching matrix and head phantom wearing a helmet interconnected to the switching matrix by means of coaxial cables.

The realized 3D microwave imaging system prototype is shown in Figure 1. It consists of several parts, described in the following sub-sections, all of which are controlled by a laptop.

#### 2.2.1. Vector Network Analyzer and Switching Matrix

First, all the signals are generated and received by a standard VNA (Keysight N5227A, 10 MHz–67 GHz) where the input power is set to 0 dBm and the intermediate frequency (IF) filter to 10 Hz. The two ports of the VNA are connected, via flexible coaxial cables, to the two input ports of the 2×24 switching matrix. The switching matrix has been realized with two single-pole-four-throw (SP4T), eight single-pole-six-throw (SP6T), and 24 single-pole-double-throw (SPDT) electro-mechanical coaxial switches. The internal connections between the switches are made with semi-rigid coaxial cables to maximize the isolation and minimize the insertion losses. Then, the 24 output ports of the switching matrix are connected, via flexible coaxial cables, to the 24 antennas placed on the helmet as supports hosting the 3D anthropomorphic head phantom. As detailed in [23], the switching matrix has been realized so that there are 24 paths from VNA port 1 to the corresponding 24 antennas, as well as 24 paths back to VNA port 2; all the paths were designed to have the same electrical length. In this way, each antenna can work as a transmitter (TX) or as a receiver (RX), and while one antenna is transmitting, the signals collected by the other 23 antennas are received in sequence by the VNA.

#### 2.2.2. Brick Antenna Array

**Figure 2:**
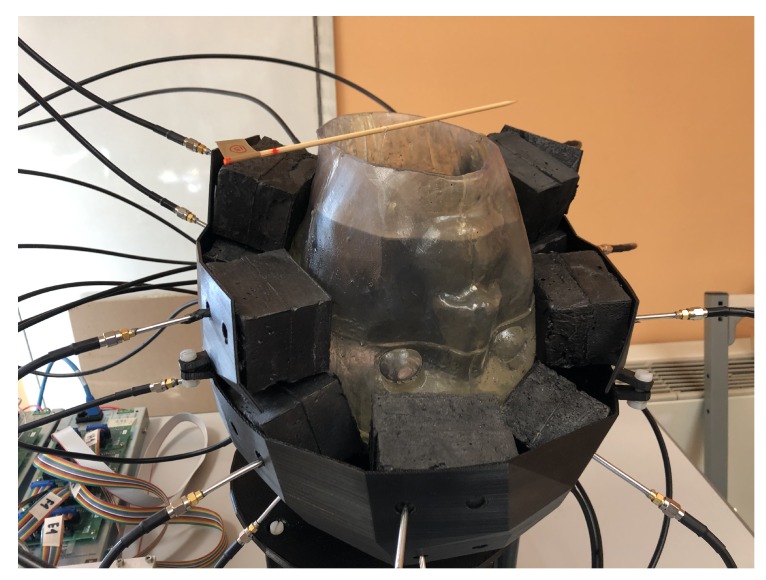
Brick antenna array conformal to the head phantom.

The antenna array is the core of the imaging system. The antenna numbers, positions and orientations were determined according to the design procedure described in Section 2.1. In the prototype shown in Figure 2, the 24 antennas are placed on a 3D printed plastic (acrylonitrile butadiene styrene, ABS) support with the shape of a helmet conformal to the head phantom. This prototype configuration allows us to easily change or remove the antennas, if needed.

Each antenna, denoted as “brick” antenna in the following, was a monopole antenna printed on a standard FR4 substrate, with a thickness equal to 1.55 mm, and embedded in a dielectric brick. The dielectric brick was made of a mixture of urethane rubber and graphite powder and was designed in order to reach a relative dielectric permittivity of $\epsilon_{r}≅20$ and to minimize the losses. The actual EM properties obtained with this mixture were $\epsilon_{r}=18.3$ and σ=0.19 S/m. The graphite powder was needed to increase the relative dielectric constant of the urethane rubber; however, it also increased the conductivity, although still within acceptable levels compared to other matching media used in medical microwave imaging (e.g., [23,24]). Moreover, the adopted matching medium is usually liquid [10], which is inherently inconvenient for a helmet-like device. Here, instead, the implemented matching medium was solid rubber, which can be easily placed conformally to the head, as shown in Figure 2. The overall dimensions of each brick were 5×5×7 cm${}^{3}$ to accommodate the need to place 24 brick antennas around the head.

**Figure 3:**
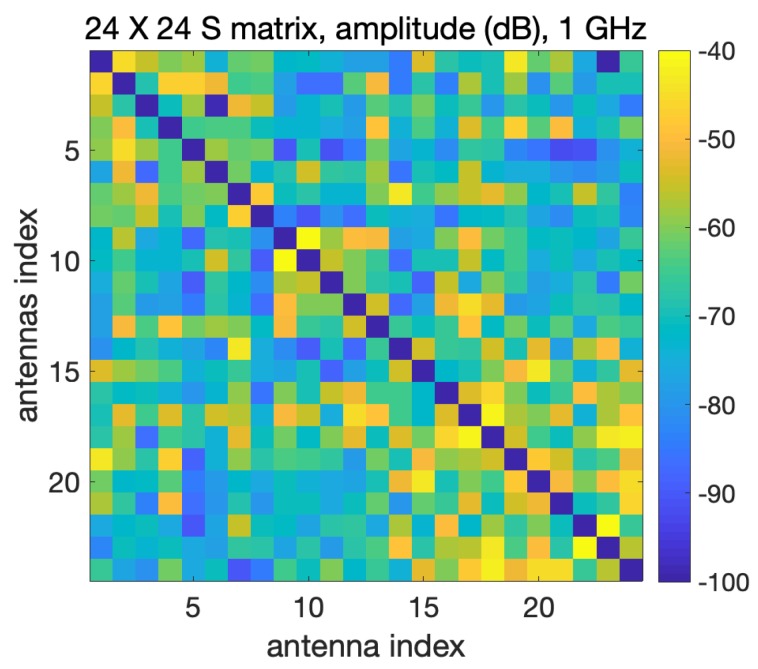
The 24×24 scattering matrix measured in the presence of the head phantom; the self-terms are forced to zero.

Figure 3 reports the 24×24 scattering matrix measured by the microwave imaging system in the presence of the head phantom; the self-terms were set to zero in order to highlight the range variation of the measured coupling coefficients, which are the input data of the used TSVD imaging algorithm.

It can be noticed that the measured signals are above −100 dB; considering that the VNA noise floor is −110 dBm (at 1 GHz and with an intermediate frequency (IF) filter equal to 10 Hz), with an input power of 0 dBm the measured data are then above the VNA noise floor [25]. Moreover, as expected, the 24×24 matrix is approximately symmetric, which confirms the reciprocity of the realized system.

#### 2.2.3. Anthropomorphic Head Phantom

The 3D anthropomorphic head phantom used for the validation and testing of the microwave imaging system was made of polyester casting resin. It was realized by additive manufacturing from a stereo-lithography (STL) file derived from MRI scans. The STL file was obtained with computer-aided design (CAD) software by modifying an original file from the Athinoula A. Martinos Center for Biomedical Imaging at Massachusetts General Hospital [26].

**Figure 4:**
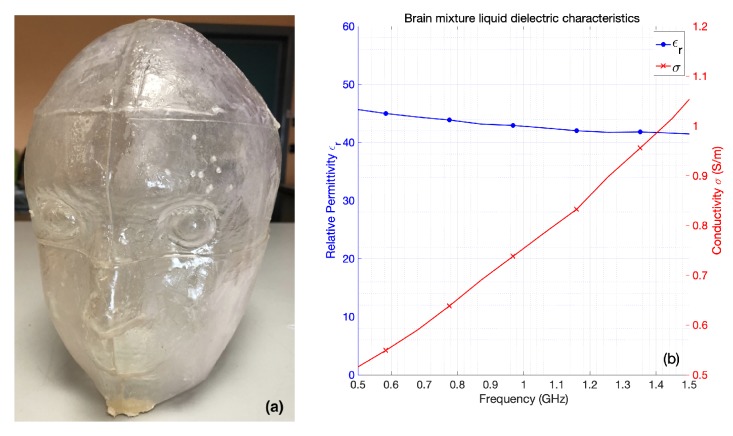
Anthropomorphic head phantom; (**a**) structure, and (**b**) dielectric characteristics of the liquid mixture, mimicking brain tissues as averages, which fills the phantom.

The phantom consisted of a cavity, shown in Figure 4a, whose wall thickness and height were equal to 3 mm and 26 cm, respectively. Its maximum cross section was approximately an ellipse whose minor and major axes were equal to 20 cm and 26 cm, respectively. The cavity was filled with a liquid mixture, made of Triton X-100, water and salt, which mimicked the average value of the dielectric characteristics of different brain tissues (white matter, gray matter) [27]. The measured dielectric characteristics of the mixture are reported in Figure 4b.

#### 2.2.4. Digital Twin of the Device

The developed device was equipped with a digital twin, namely an accurate full-wave numerical model simulating its behavior, thanks to the use of proper CAD and EM simulation softwares. This tool had a two-fold purpose. First, it allowed us to foresee the expected outcomes of the planned experiments and to analyze them before running the actual experiments. Second, it provided the EM fields $E_{b}$ needed to build the imaging kernel, as described in Section 2.1.

**Figure 5:**
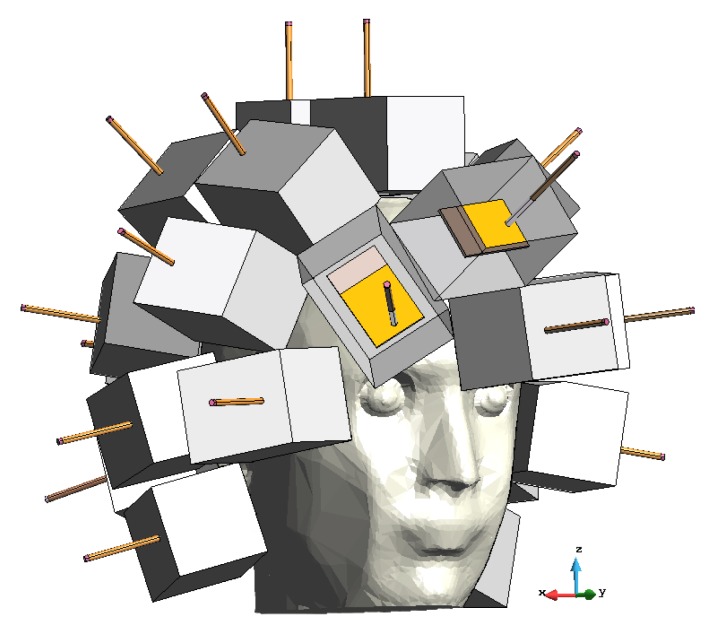
Computer-assisted design (CAD) model of the 3D microwave imaging system together with the anthropomorphic head phantom; two printed monopole antennas within the coupling medium bricks are highlighted in yellow.

Figure 5 shows the CAD model of the device which includes both the antenna array and the 3D anthropomorphic head phantom.

Each brick, placed on the head, represented one TX/RX antenna together with the dielectric coupling medium; the antenna ports were placed at the end of the coaxial cables leading out from the bricks. The dielectric characteristics of the bricks were the same as the nominal ones used in the realized system (see Section 2.2.2), i.e., $\epsilon_{r}=18.5$ and σ=0.2 S/m. The head phantom was made of a dielectric medium representing the average brain tissues with $\epsilon_{r}=42.5$ and σ=0.75 S/m, according to the properties of the medium adopted in the experimental validation (Section 2.2.3).

To perform the EM simulation, which provided both the S-parameters at the antenna ports and the EM fields in the whole scenario, the CAD model was introduced into an in-house full-wave software, based on the finite element method (FEM) [28]. The implemented software used the standard “curl–curl” formulation for the electric field and Galerkin testing. The whole volume, including the CAD model, was discretized with edge-basis functions, defined over a mesh of tetrahedral cells. The antenna metal parts were modeled as perfect electric conductors (PEC), and all the dielectrics were modeled via sub-volumes with given $\epsilon_{r}$ and σ values. The tetrahedral mesh was terminated at the volume boundaries with appropriate absorbing boundary conditions (ABC). Each antenna port was modeled as a coaxial cable section where, if excited, a tangential electric field distribution was enforced.

In the numerical modeling of the MWI system, the 24 antennas were excited one at a time, setting all the others as receivers in order to generate the 24×24 scattering matrix. Moreover, the field radiated by each antenna was evaluated at different locations within the head to generate the discretized scattering operator S in (1).

#### 2.2.5. Experiment Set-Up

**Figure 6:**
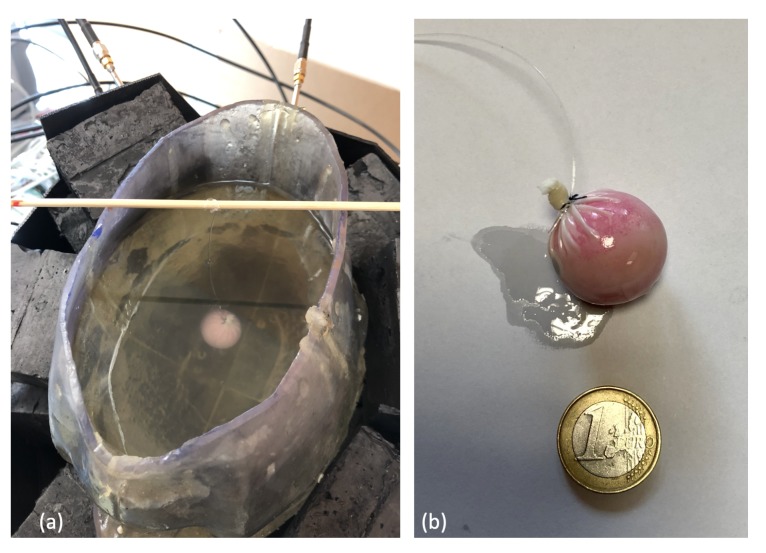
(**a**) Experimental set-up; (**b**) 1.25 cm-radius plastic sphere.

The microwave imaging system was validated using the 3D anthropomorphic head phantom in which a 1.25 cm-radius plastic sphere was inserted, as shown in Figure 6.

**Figure 7:**
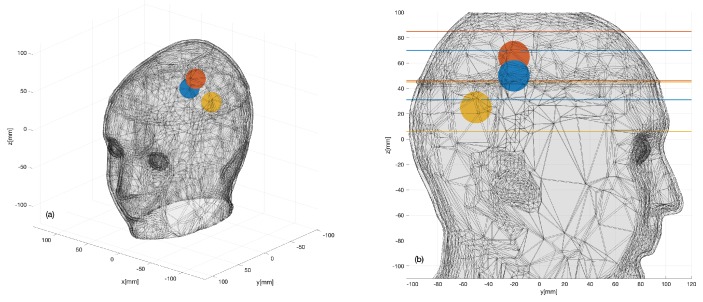
Three sphere locations (color dots) considered within the 3D head phantom: (**a**) 3D view; (**b**) 2D sagittal view.

The sphere was fixed via a monofilament polymer line (fishing line) to an external support located above the head and was immersed in the liquid mixture at a location and height that could be varied. The sphere density was higher than the liquid mixture filling the head phantom, and the sphere was therefore not floating on the liquid and could be easily positioned in different locations within the head phantom. Three positions were considered, as shown in Figure 7. However, as their exact location could be affected by inaccuracies, it is obvious that the actual positions may have slightly differed from the expected ones.

The aim of these experiments was to identify the 3D shape and location of the sphere that was supposed to represent the region of the brain affected by the stroke. In this respect, it is important to remark that, while the plastic sphere adopted here for the sake of simplicity was not intended to mimic a hemorrhage or a clot, it showed a dielectric contrast with brain tissues which was comparable to a hemorrhage but with the opposite sign. Thus, the experiment was expected to provide a meaningful, though initial, validation of the imaging capabilities of the prototype device.

## 3. Results

**Figure 8:**
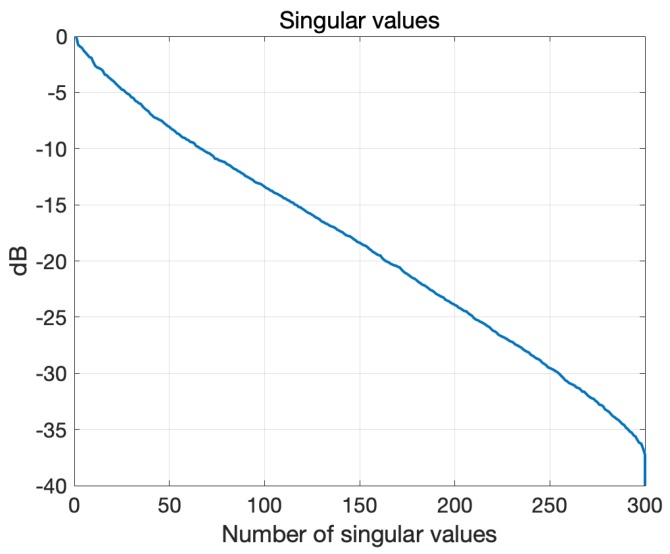
Singular value behavior of the scattering operator.

In the following, we describe the validation of the realized 3D microwave imaging system (Section 2.2), whose experimental set-up is detailed above in Section 2.2.5. First, by means of the digital twin, the outcomes of the experiments are foreseen and assessed. Then, the results of the actual experiments are reported. All the imaging results were obtained by using the TSVD algorithm described in Section 2.1. Figure 8 shows the singular values calculated for the relevant scattering operator, which were computed with the digital twin. The truncation index $L_{t}$ in (2) was set to −20 dB for all the reported cases. To quantitatively assess the quality of the reconstruction images, the root mean square error (RMSE) was evaluated as

(3) $$\mathrm{RMSE}=\sqrt{\frac{\sum_{n=1}^{N_{s}}{\left(\Delta \hat{\chi }-\Delta \chi \right)}^{2}}{N_{s}}},$$

where $N_{s}$ is the number of samples of the discretized domain, $\Delta \hat{\chi }$ the retrieved differential contrast and Δχ is the actual contrast.

### 3.1. Numerical Assessment

**Figure 9:**
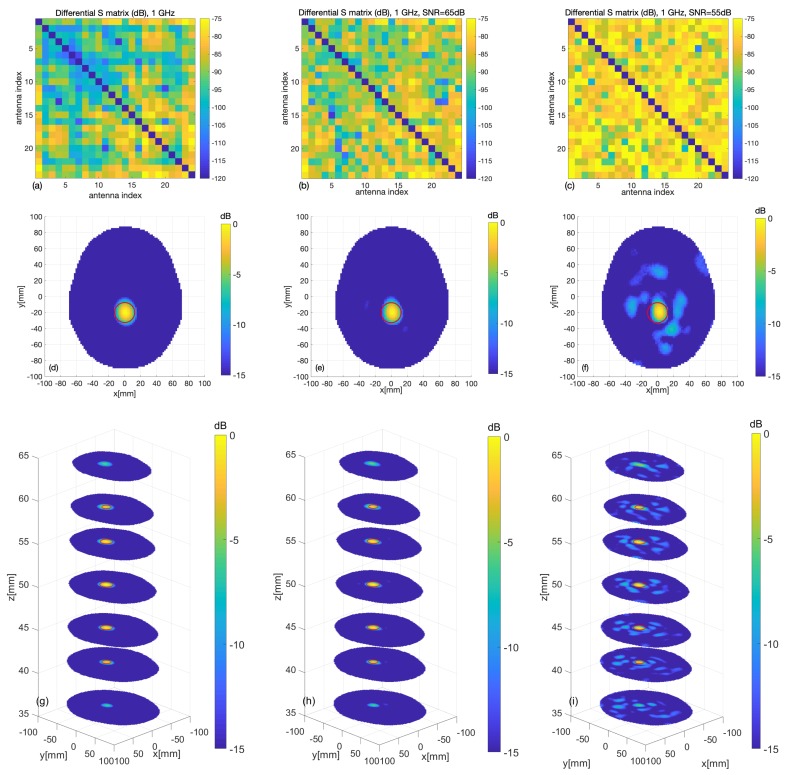
Digital twin: case of a target plastic sphere; the exact sphere location and shape are indicated by red circles; (**a**–**c**) differential scattering matrices, and (**d**–**f**) cross-sections of the reconstructed images at the sphere center and (**g**–**i**) at various levels, for different values of the signal-to-noise ratio (SNR) (left column: no noise, middle column: SNR = 65 dB, right column: SNR = 55 dB).

To confirm the validity of the above statement, the digital twin of the system described in Section 2.2.4 was exploited. To this end, the experiment for the target corresponding to the blue sphere in Figure 7 was simulated by including a 1.25 cm-radius plastic sphere with $\epsilon_{r}=2.1$ in the CAD head phantom. The simulation was repeated for the case of noiseless measurements (to provide an ideal benchmark) and for two SNR levels, namely 65 dB and 55 dB. Noise was modeled as an additive Gaussian white noise, which was added to the simulated data given by the S matrices computed with and without the target, respectively. Figure 9 shows the outcomes of the simulations. In particular, the first row shows the amplitude of the scattering matrices, where, in agreement with the actual experimental situation, the self-contributions have been omitted. It appears that the considered noise level severely affects the data matrix. The middle and bottom rows of Figure 9 show the normalized amplitude of the reconstructed differential contrast Δχ arising from the TSVD algorithm. It is worthwhile to note that the target is properly imaged, even when the SNR is comparable to or higher than the maximum values of the differential S matrix and the corresponding matrices appear very noisy (see Figure 9b,c), as the unavoidable occurrence of some artifacts does not restrict the interpretation of the result. The RMSE values obtained for the reconstructions at the three considered noise levels are 0.04, 0.06 and 0.11, respectively.

**Figure 10:**
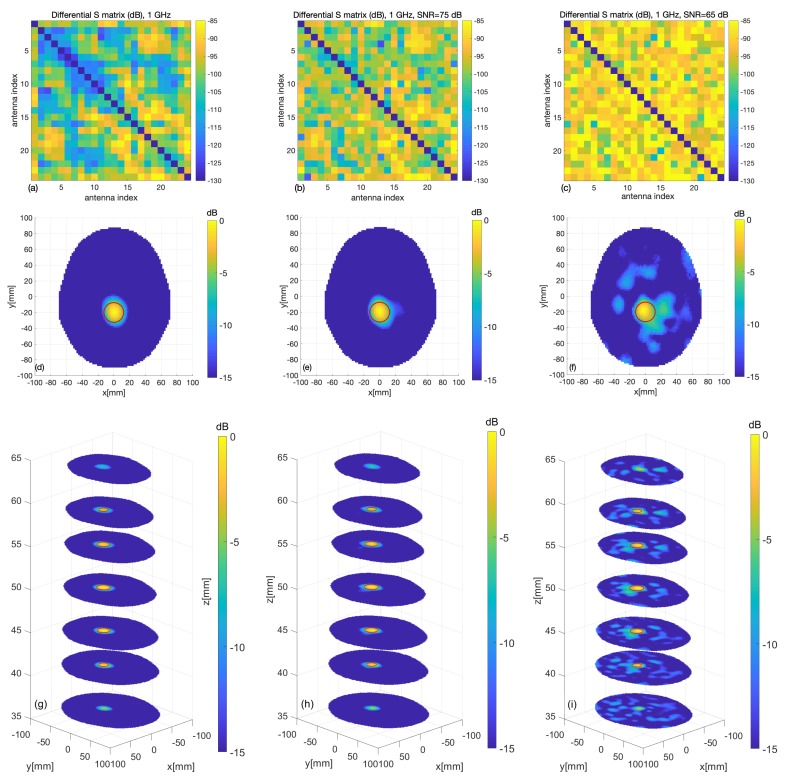
Digital twin: case of a target blood sphere; the exact sphere location and shape are indicated by red circles. (**a**–**c**) Differential scattering matrices; (**d**–**f**) cross-sections of the reconstructed images at the sphere center and (**g**–**i**) at various levels, for different values of the signal-to-noise ratio (left column: no noise, middle column: SNR = 75 dB, right column: SNR = 65 dB).

Once the kinds of results that the device was expected to provide in the experiments had been characterized, the second goal was to show whether—and to what extent—the experiment was relevant for the considered clinical scenario. To this end, the same simulation as before was repeated considering a spherical target with the properties of blood ($\epsilon_{r}=63.4$ and σ=1.6 S/m) instead of plastic, thus mimicking a hemorrhagic stroke. The results of the simulations are shown in Figure 10. Figure 10d–i show the imaging results, which are comparable with the previous ones but for an expected increased presence of (not meaningful) artifacts. In agreement with this, the RMSE values are essentially the same as for the case of the plastic target, at 0.04, 0.06 and 0.13 for the different considered levels of noise, respectively. According to the above results, we can conclude that the developed prototype will be able to pass the planned experimental validation and that the considered experiments provide a relevant, though initial, test-bed for the designed microwave imaging system.

### 3.2. Experimental Validation

To perform the experimental validation of the system, for each sphere location, two measurement sets were taken at different times. The first data-set was measured in the absence of the sphere, and the second one was taken when the sphere was positioned inside the phantom. Each pair of measured 24×24 scattering matrices were then differentiated and given as an input to the TSVD algorithm. It can be observed that VNA port calibration was not needed as possible systematic measurement errors were cancelled out via the use of differential data or did not affect the obtained qualitative imaging of the differential contrast function. The time needed to perform the total measurement with the current prototype was less than 4 min for each sphere location, and the elapsed time between the two measurement sets was around 10 min. The kernel simulated with the digital twin (Section 2.2.4) was exploited to generate the images, and the data processing time needed by the TSVD algorithm was negligible (less than 1 s).

**Figure 11:**
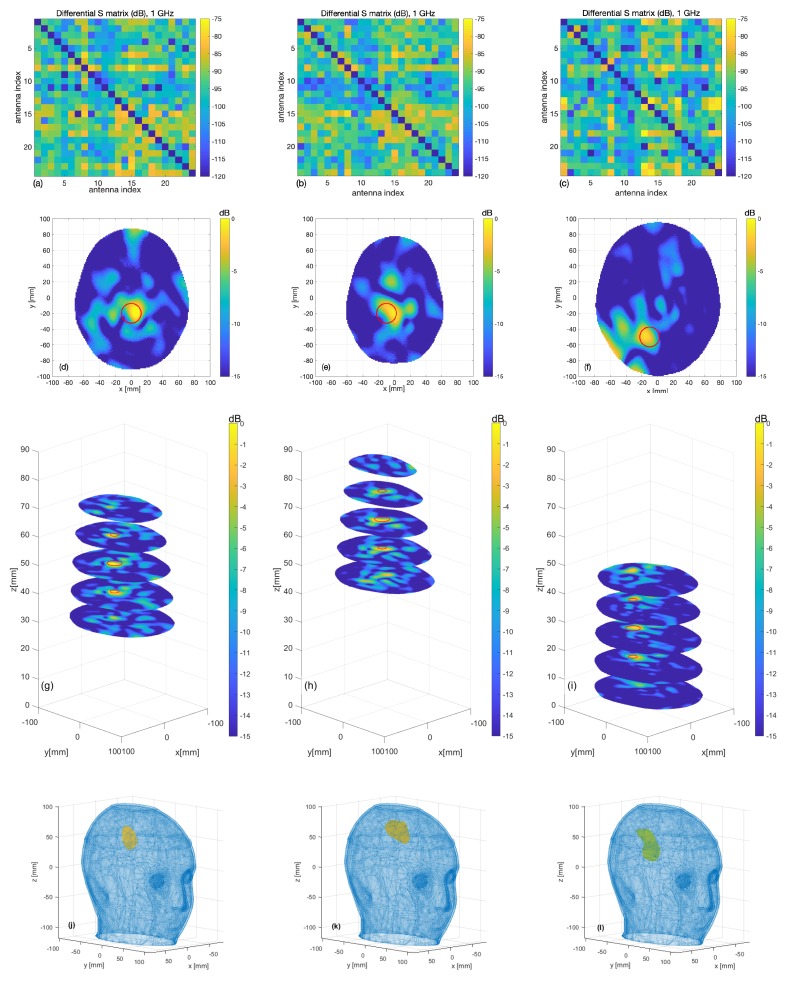
Measurement results: (**a**–**c**) differential scattering matrices; (**d**–**f**) horizontal cross-sections at sphere center and (**g**–**i**) at the various levels as highlighted in Figure 7 (the expected sphere location and shape are indicated by red circles); from left to right, images corresponding to the blue, red and yellow spheres of Figure 7, respectively; (**j**–**l**) the 3D rendering of the imaged stroke.

**Figure 12:**
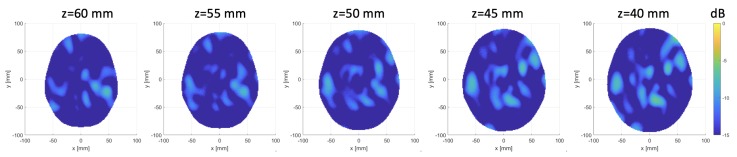
Horizontal cross-sections at various levels of the obtained 3D image, differentiating two sets of different measurements of the same scenario; images normalized with respect to the maximum value of Figure 11b.

The 24×24 differential scattering matrices obtained for the three cases are shown in Figure 11a–c. The second and third rows of Figure 11 display the images obtained for the different sphere locations indicated in Figure 7. The second row corresponds to the horizontal cross-section passing through the sphere center; the expected sphere location and size are highlighted with a red circle. The third row displays horizontal cross-sections at the different levels indicated in Figure 7b. In all cases, the targets are very well retrieved, and the images agree with the simulation results, which confirms our expectations. The RMSE values for these reconstructions are 0.16, 0.19 and 0.18 for the three considered positions of the target, respectively. Moreover, the last row of Figure 11 shows the 3D rendering of the imaged stroke obtained by plotting the values of the normalized differential contrast amplitude, which are above −3 dB. Finally, Figure 12 reports horizontal cross-sections at various levels of the 3D reconstructed image obtained by differentiating two different sets of measurements of the same scenario; all the images are normalized with respect to the maximum value of Figure 11b, which represents a case when the target is present.

## 4. Discussion

The main goal of the developed device was to image (qualitatively) possible anomalies (clots or hemorrhages) in the head to support clinicians in the evaluation of the effectiveness of the administrated therapies. The results shown in the previous section, although preliminary, confirm the potential of the technology in providing reliable results, as it is capable of imaging a target as small as 1.25 cm in radius.

A second important achievement of the analysis carried out here is represented by the validation of the proposed system through its digital twin, which provides simulated data which are quantitatively consistent with the measured data. As such, the adopted modeling tool provides a reliable representation of the device, making it possible both to build the imaging kernel and to synthetically reproduce laboratory experiments. As a matter of fact, the results from the simulations and measurements appear to be essentially the same, except for a slight deterioration of the images in the case of measured data. In particular, the worse RMSE results in the experimental tests, with respect to the corresponding numerical cases, are due to the possible inaccuracies in the expected positions of the target as well as to model inaccuracies in the digital twin.

A very important feature of the presented device is its robustness against false positives, assessed through a specific experiment, the result of which is shown in Figure 12. We can observe that the reconstructed values, which represent only the overall noise between different sets of measurements, are significantly lower than 0 dB (the maximum value in the 3D reconstructed image is equal to −4.75 dB) and are therefore clearly different from the cases when the target is present (see Figure 11).

To the best of our knowledge, this paper presents the first system based on a low-complexity antenna arrangement conformal to the head which is able to provide full 3D images; other imaging systems available in the literature only provide 2D images and often exploit a large number of antennas.

With this work representing a preliminary validation of the developed hardware, it is worthy of note that, for practical reasons, the target used in the experimental test does not exhibit the same dielectric properties as a stroke. On the other hand, the digital twin can help us to predict what will happen in an experiment dealing with a target mimicking a hemorrhage, for example. As a matter of fact, by comparing results from simulations and measurements, it can be observed that the differential scattering matrices exhibit a similar pattern but are lower in amplitude (by about 10 dB) in the case of simulations due to the lower maximum amplitude of the differential contrast between blood and the average brain with respect to plastic and the average brain (0.49 and −0.95, respectively). This implies that, in an experiment dealing with a target mimicking a hemorrhage, slightly weaker useful signals should be collected. However, this is not a significant limitation, as the amplitudes of the differential scattering matrices are well above the VNA noise floor, which represents the ultimate limitation for accurate measurements [25].

Finally, while the repeatability of the experiment has not been tested in this paper with respect to the possible misalignment of the phantom in the two gathered data sets, from our previous studies, we expect that such uncertainties will produce “structured” artifacts in the final image, which are easily attributable to positioning errors [19].

## 5. Conclusions and Future Work

In this paper, a prototype of a novel low-complexity device, dedicated to brain stroke monitoring in the post-acute stage, has been presented. The reported experiments aimed to provide an initial validation of the device and confirm that there is an agreement between the design of the system [18] and its performance. This is assessed by the comparison of the achieved results with those obtained from its digital twin. The availability of such a model also allows us to show the kinds of outcomes that are expected to be obtained by the system when operated with more realistic targets.

The next steps of the system validation and development will involve an assessment with the more realistic anthropomorphic head phantom described in [27], which includes an additional cavity modeling a stroke [29]. As this phantom is derived from an STL file, a numerical validation using the digital model of the system will also be carried out in this case. In addition, improvements of the prototype will be performed by considering image reconstruction procedures using multi-frequency data calibration techniques as well as sparsity-promoting regularization schemes; furthermore, there will be a refinement of the antenna array support to make it wearable.

## Abbreviations

The following abbreviations are used in this manuscript:

MRI	Magnetic resonance imaging
CT	Computerized tomography
MWI	Microwave imaging
VNA	Vector network analyzer
EM	Electromagnetic
SNR	Signal-to-noise ratio
TSVD	Truncated singular value decomposition
SP4T	Single-pole-four-throw
SPDT	Single-pole-double-throw
IF	Intermediate frequency
TX	Transmitter
RX	Receiver
ABS	Acrylonitrile butadiene styrene
STL	Stereo-lithography
FEM	Finite element method
PEC	Perfect electric conductor
ABC	Absorbing boundary conditions
CAD	Proper computer-aided design
RMSE	Root mean square error
